# Risk of Dengue for Tourists and Teams during the World Cup 2014 in Brazil

**DOI:** 10.1371/journal.pntd.0003063

**Published:** 2014-07-31

**Authors:** Willem G. van Panhuis, Sangwon Hyun, Kayleigh Blaney, Ernesto T. A. Marques, Giovanini E. Coelho, João Bosco Siqueira, Ryan Tibshirani, Jarbas B. da Silva, Roni Rosenfeld

**Affiliations:** 1 University of Pittsburgh Graduate School of Public Health, Pittsburgh, Pennsylvania, United States of America; 2 Carnegie Mellon University, Pittsburgh, Pennsylvania, United States of America; 3 University of Pittsburgh Center for Vaccine Research, Pittsburgh, Pennsylvania, United States of America; 4 Brazil Ministério da Saúde, Brasilia, Departamento Federal, Brazil; 5 Universidade Federal de Goiás, Goiânia, Goiás, Brazil; Centers for Disease Control and Prevention, United States of America

## Abstract

**Background:**

This year, Brazil will host about 600,000 foreign visitors during the 2014 FIFA World Cup. The concern of possible dengue transmission during this event has been raised given the high transmission rates reported in the past by this country.

**Methodology/Principal Findings:**

We used dengue incidence rates reported by each host city during previous years (2001–2013) to estimate the risk of dengue during the World Cup for tourists and teams. Two statistical models were used: a percentile rank (PR) and an Empirical Bayes (EB) model. Expected IR's during the games were generally low (<10/100,000) but predictions varied across locations and between models. Based on current ticket allocations, the mean number of expected symptomatic dengue cases ranged from 26 (PR, 10^th^–100^th^ percentile: 5–334 cases) to 59 (EB, 95% credible interval: 30–77 cases) among foreign tourists but none are expected among teams. These numbers will highly depend on actual travel schedules and dengue immunity among visitors. Sensitivity analysis for both models indicated that the expected number of cases could be as low as 4 or 5 with 100,000 visitors and as high as 38 or 70 with 800,000 visitors (PR and EB, respectively).

**Conclusion/Significance:**

The risk of dengue among tourists during the World Cup is expected to be small due to immunity among the Brazil host population provided by last year's epidemic with the same DENV serotypes. Quantitative risk estimates by different groups and methodologies should be made routinely for mass gathering events.

## Author Summary

This year the 2014 FIFA World Cup will be hosted by Brazil, a country that has reported a higher number of dengue cases annually than any other country worldwide over the last decade. About 600,000 foreign tourists are expected and may be at risk for this disease. Games will be played in 12 different cities across the country and teams will stay in 27 different basecamp locations. We used weekly dengue surveillance data from previous years (2001–2013) and the first 19 weeks in 2014 to estimate the risk of dengue during the World Cup in each location and found that the expected incidence rates were relatively low. We also found interesting differences across estimation methods. Based on current ticket allocations, we expect that between 26 and 59 dengue cases will occur among tourists and none among teams. Quantitative risk estimates based on historical data should be made routinely for mass gathering events.

## Introduction

Dengue is a mosquito-borne viral disease predominantly spread by female *Aedes aegypti* mosquitoes in (sub) tropical regions of the world. The dengue virus (DENV) causes an estimated 390 million infections per year worldwide resulting in 96 million clinically symptomatic cases [1]. The DENV has four serotypes (DENV-1, DENV-2, DENV-3, and DENV-4). Infection with DENV provides lifelong immunity against the infecting serotype and cross-protection against the other serotypes that lasts for about two years [2]. Secondary infection with a heterologous serotype is thought to increase the risk of dengue hemorrhagic fever (DHF) – the severe form of dengue – and death [3].

During the past years, Brazil has reported more dengue cases than any other country with over half a million cases per year since 2007 and almost one million cases in 2010 [4].

This year, Brazil will host one of the largest sport events worldwide: the 2014 FIFA World Cup. Football teams from 32 countries across every continent and their supporters will travel to Brazil during the months of June and July. A total of 64 games will be played in 12 cities across the country. A total of 2.5 million tickets will be sold and it is expected that between 300,000 and 600,000 supporters from countries outside Brazil will visit the games [5],[6],[7].

Large scale events such as the World Cup have raised concern about the possible spread of infectious diseases among visiting tourists and the local population. In the past, outbreaks have occurred during major sports events. Measles epidemics occurred during the Special Olympics in Minneapolis-St. Paul in 1991 [8] and during the 2010 World Cup in South Africa [9]. Norovirus outbreaks have been reported during the 2006 World Cup in Germany [10] and a Scout Jamboree in The Netherlands [11]. In response to this possible threat, international and national health agencies have developed specific plans for disease surveillance and response during these mass gathering events [12]. The possible risk of dengue among tourists visiting the 2014 World Cup in Brazil has been mentioned before [10],[13],[14]. Between 1997 and 2013, dengue was the most common vector-borne disease among 1586 travelers returning from Brazil with 92 cases reported to the GeoSentinel Network [14],[15].

DENV transmission rates and seasonality vary substantially across Brazil, resulting in large variation in the risk of dengue during the World Cup in game and team basecamp cities that are spread across the entire country. This heterogeneity has implications for the distribution of risk among tourists and teams visiting from various countries. We used historical dengue surveillance data for game and basecamp cities to estimate the risk of dengue during the World Cup weeks for tourists and teams. We found that this risk was low but varying across locations.

## Methods

### Data

We used historical dengue surveillance data to estimate the risk of symptomatic dengue infection during the World Cup. This risk was estimated separately for game cities visited by tourists and basecamp cities where country teams will reside (**Table S1**). Weekly dengue incidence rates (IR's) from 2001 to 2013 were available for all cities. Weekly IR's for the first 19 weeks of 2014 were also available for game cities but not for basecamp cities. All data have been provided by the Brazil Ministry of Health and have been collected by a passive dengue surveillance system. We used confirmed (by laboratory testing or epidemiological links) dengue cases in this analysis. Because the surveillance system only captured symptomatic cases among the Brazil population, we estimated the risk of symptomatic cases in this analysis. Since we only used already collected, aggregated dengue surveillance data that does not identify any individuals, this study is exempt from human subject research.

### Estimation of risk

We estimated the risk of dengue in game and basecamp cities per week of the World Cup. The World Cup will consist of two rounds. During the first round, each team will play three qualifying games between June 12^th^ and 26^th^ (weeks 24–26). The location of each of these games is already known (**Table S1**). During the second round (June 28^th^ -July 13^th^ or weeks 27–29), 16 qualified teams will compete for the final. We estimated the risk of dengue separately for each round. Country teams will arrive one week before the games and their first round will be one week longer (weeks 23–26) compared to tourists.

For each city, we used two methods to forecast the 2014 IR's during the World Cup weeks. Both methods were based on IR's of preceding years (2001 to 2013). First, we used a percentile rank (PR) method. For game cities, we computed the percentile of IR's in weeks 2014 1-19 on the distribution of the corresponding weeks in 2001–2013. We used a weighted average of these percentiles as indicator of the severity of the 2014 dengue season compared to the previous years (P2014). For this average, the percentile of each week in 2014 (1 through 19) was weighted by the week number, i.e. week one was given a weight of one and week nineteen was given a weight of nineteen. This allowed a greater contribution of weeks closer to the World Cup to the predicted IR's. We then estimated IR's during the World Cup weeks 23–29 by taking the P2014 of the 2001–2013 distribution for each of these weeks (IR_P2014_). For basecamp cities, 2014 data were not available and we used the average P2014 of all game cities within a 600 km radius from each basecamp as an estimate of the P2014 for basecamps. To indicate uncertainty in our estimates, we also computed IR's during World Cup weeks based on the 10^th^ percentile (P10) and the maximum of the 2001–2013 distribution.

Secondly, we developed an Empirical Bayes (EB) model of previous (2001–2013) DENV epidemics in the 12 games cities compared to 2014 rates. This method could not be used for basecamp cities given that 2014 data were not available for these cities. Empirical Bayes uses historical data to form the prior distribution of model parameters, which is different from conventional Bayes methods that use a fixed prior distribution. EB assumes that the coming season will resemble one of the past seasons in the same locality, but allow variation in epidemic magnitude, timing, and duration, as well as added random fluctuations. We computed a prior distribution for each game city consisting of the observed epidemics in 2001–2013 and of added variations of these epidemics by shifting in timing with −2 to +2 weeks and in amplitude with multiplication factors ranging from 0.75 to 1.25. We used the 2014 weeks 1–19 and a Gaussian Noise model of these data to yield a posterior distribution for likely IR trajectories during the remaining weeks in 2014. From this posterior distribution, we computed the mean and pointwise 95% Baysian credible intervals for IR's during the World Cup weeks. See **Supporting Text 1** for more detail on the Empirical Bayes model.

We determined the expected IR's for tourists per country of origin by averaging the IR's for each of the game cities for their team during round one of the World Cup. No country specific estimates could be made for round two since the game locations are still unknown.

### Estimation of potential dengue cases

We used estimated IR's during World Cup weeks to compute the number of symptomatic dengue cases expected among tourists and teams. Between 300,000 and 600,000 foreign visitors are expected during the World Cup and approximate distributions of tickets among the top ten countries with the most tickets allocated have been released by the Fédération Internationale de Football Association (FIFA) [5]–[7]. We assumed that 600,000 foreign visitors will visit the World Cup and that they will be distributed among countries according to ticket allocations (**Table S2**). We distributed the difference between 600,000 and the 484,237 total allocated tickets equally among the participating countries for which no ticket allocations have been published. Given that the number of dengue cases depends on the local risk in each game city and that it will remain unknown where each country team will play until completion of round one, we assumed that all tourists will visit during round one only. We distributed the number of visitors from each country equally across their three game cities during round one and computed the number of cases for each city using the expected IR's for each week in this round. We assumed that visitors will stay for an average of two weeks based on information on tourists that have visited Brazil in the past [16]. We computed the number of cases separately for IR's estimated with the PR or the EB method. For each country, the number of cases was first computed per game city and per week, and then aggregated before rounding the number of cases to whole digits. The rounded numbers of cases were aggregated for the total number of cases expected across all countries. In addition to estimating the number of cases for 600,000 tourists, we also conducted a sensitivity analysis of 100,000 to 800,000 tourists. In addition to tourists, we estimated the number of dengue cases that would be expected among teams of 23 players staying at their basecamps for the full duration of the Word Cup. For teams, we also computed the number of cases for a size of 500 (e.g. including staff, family, media, etc.).

## Results

### Dengue transmission in the past

**Figure 1:**
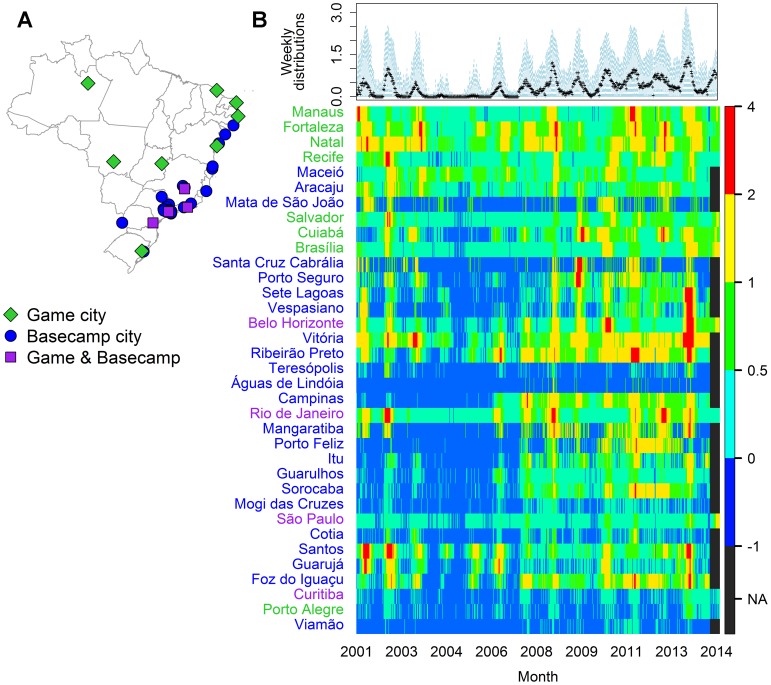
Weekly log 10 incidence rates from 2001 to 2013 for cities that host games, teams (basecamps), or both. (**A**) Locations of World Cup cities, (**B**) Log 10 incidence rates per week by city with the distribution of all weekly incidence rates across cities in the top panel.

Dengue IR's varied substantially across game and basecamp cities during 2001–2013 (**Figure 1**). From 2001–2007, transmission was highest in northern cities on the East Coast and virtually absent in southern cities except during the 2002 epidemic. After 2007, DENV transmission increased across all cities with one of the largest epidemics occurring last year (2013) with IR's exceeding 1500 cases/100,000 in some cities.

### Risk of dengue during the World Cup

**Figure 2:**
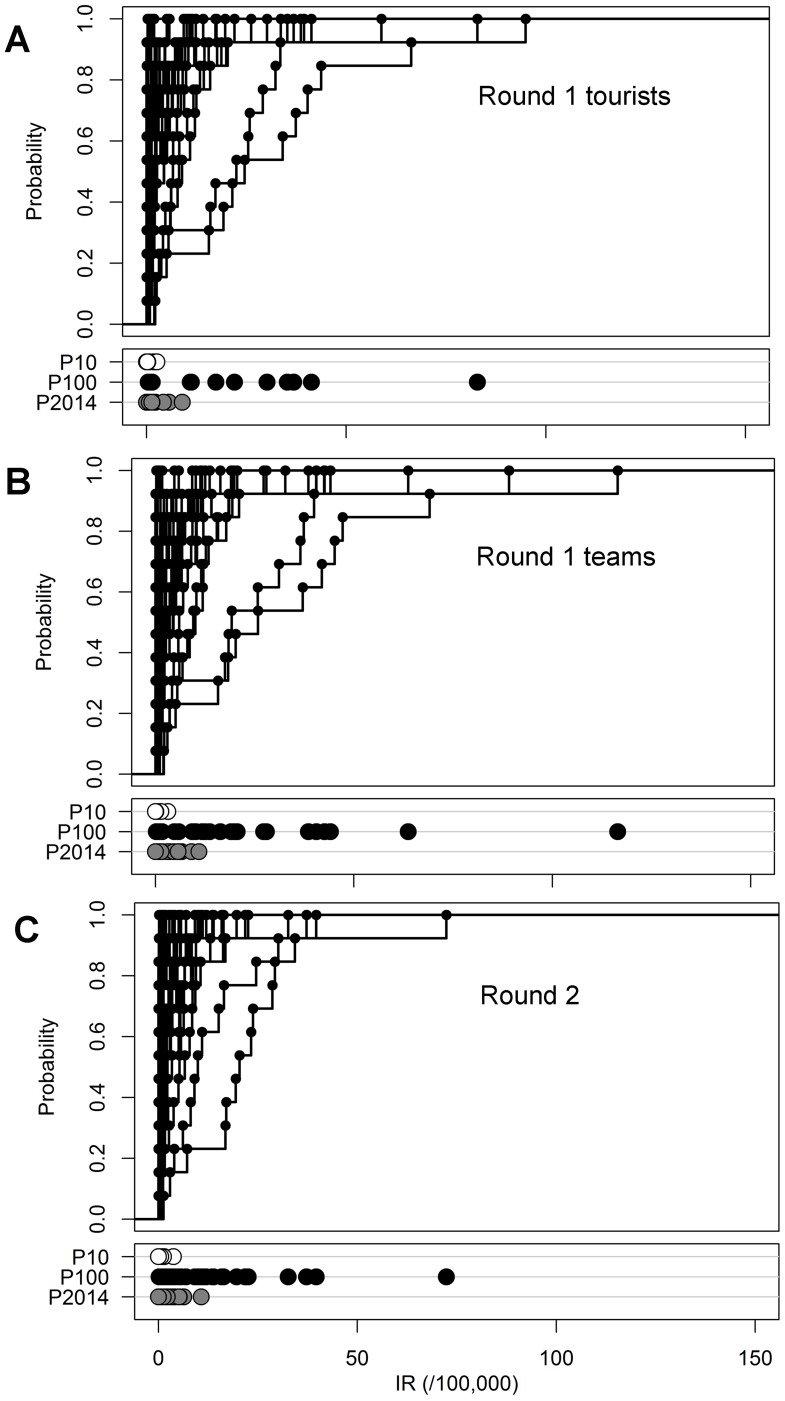
Probability of weekly incidence rates based on the 2001–2013 distribution for each city. Probabilities are shown for (**A**) round one for tourists, (**B**) round one for teams, and (**C**) round two of the World Cup. The 10^th^ percentile (P10), maximum (P100) and 2014 percentile (P2014) of the 2001–2013 distribution are indicated for each city in the lower panels.

We used two methods to forecast expected IR's during 2014: percentile rank (PR) and Empirical Bayes (EB). The percentile of weeks 1–19 in 2014 on the distribution of corresponding weeks in 2001–2013 (P2014) was highest for São Paulo at 96%, indicating that the current 2014 epidemic has been one of the worst in it's history (**Figure S1**). For most other cities, the P2014 ranged from 20% to 65%. For each city, the probability distributions of the average weekly IR's during World Cup weeks in previous years were estimated separately (**Figures 2A-C** **and S2**). The cities of Fortaleza and Natal had consistently higher past and expected IR's compared to all other cities with an IR_P2014_ in round one of 5.6 cases/100,000 (P10-Max: 1.8–35.3) and 8.9 cases/100,000 (P10-Max: 2.6–82.9) respectively. For round two, their IR_P2014_ was 6.4 cases/100,000 (P10-Max: 1.4–37.2) and 10.8 cases/100,000 (P10-Max: 3.8–72.4) respectively (**Table 1**). For all cities, the maximum IR's reported in previous years were substantially higher compared to the IR_P2014_ due to some large epidemics that occurred in the past. For example the maximum IR for the basecamp city of Santos (116.5/100,000) was 25 times as high as the IR_P2014_ for this city (4.6/100,000). These maximum IR's represent the worst case scenario based on previous epidemics.

**Table 1:** Weekly estimated incidence rates (/100,000) per city during the World Cup.

City	Team*	Round 1†			Round 2‡		
		IR_P2014_	P10	Max.	IR_P2014_	P10	Max.
**Games/tourists**							
Manaus	-	2.25	0.62	11.22	2.20	0.61	6.96
Fortaleza	-	5.63	1.82	35.29	6.39	1.44	37.25
Natal	-	8.94	2.62	82.86	10.77	3.77	72.38
Recife	-	1.73	0.21	41.32	1.74	0.23	39.65
Salvador	-	4.25	0.30	10.95	4.04	0.25	9.84
Belo Horizonte	-	1.41	0.10	22.04	0.44	0.03	5.69
Rio de Janeiro	-	0.38	0.10	36.86	0.32	0.12	13.90
São Paulo	-	0.94	0.04	1.44	0.71	0.04	1.07
Curitiba	-	0.02	0.00	0.42	0.00	0.00	0.32
Porto Alegre	-	0.02	0.00	0.53	0.00	0.00	0.14
Cuiabá	-	0.69	0.01	30.21	0.68	0.00	16.35
Brasília	-	1.34	0.23	17.35	0.96	0.13	11.11
**Basecamps/teams**							
Belo Horizonte	CHI	1.76	0.17	27.95	0.44	0.03	5.69
Rio de Janeiro	ENG, NED	0.41	0.10	42.64	0.32	0.12	13.90
São Paulo	USA	1.08	0.04	1.64	0.71	0.04	1.07
Curitiba	ESP	0.03	0.00	0.43	0.00	0.00	0.32
Maceió	GHA	5.8	1.14	40.55	5.23	0.70	21.84
Aracaju	GRE	3.49	1.10	11.78	2.34	1.07	12.02
Mata de São João	CRO	6.61	0.00	19.05	3.20	0.00	9.15
Porto Seguro	SUI	5.92	0.03	9.28	3.46	0.05	4.07
Santa Cruz Cabrália	GER	1.73	0.00	4.76	1.21	0.00	5.08
Sete Lagoas	URU	1.56	0.05	16.37	1.05	0.00	3.51
Vespasiano	ARG	1.44	0.00	20.60	0.39	0.00	1.79
Vitória	AUS, CMR	9.11	3.15	63.69	5.13	1.28	32.64
Mangaratiba	ITA	3.08	0.00	12.56	1.38	0.00	10.47
Teresópolis	BRA	0.15	0.00	0.60	0.00	0.00	0.60
Águas de Lindóia	CIV	0.00	0.00	1.44	0.00	0.00	1.91
Campinas	NGA, POR	5.73	0.00	10.18	3.00	0.00	5.68
Cotia	COL	0.13	0.00	0.95	0.12	0.00	0.50
Guarujá	BIH	1.49	0.1	44.13	0.64	0.11	10.54
Guarulhos	IRN	0.77	0.02	1.71	0.48	0.00	1.45
Itu	JPN, RUS	1.54	0.00	5.88	0.54	0.00	2.75
Mogi das Cruzes	BEL	0.18	0.00	0.54	0.00	0.00	0.43
Porto Feliz	HON	0.50	0.00	19.84	0.00	0.00	19.67
Ribeirão Preto	FRA	10.98	0.36	38.54	5.18	0.22	15.90
Santos	CRC, MEX	4.6	1.36	116.50	2.25	0.59	22.58
Sorocaba	ALG	1.43	0.01	27.28	0.85	0.00	13.59
Foz do Iguaçu	KOR	5.72	0.11	13.67	1.22	0.00	10.28
Viamão	ECU	0.00	0.00	0.21	0.00	0.00	0.56

*Country ISO code, † Round 1 for teams is one week longer as teams arrive a week earlier.

‡ Round 2 is one week of games, a semifinal in Sao Paulo, and a final in Rio de Janeiro.

**Figure 3:**
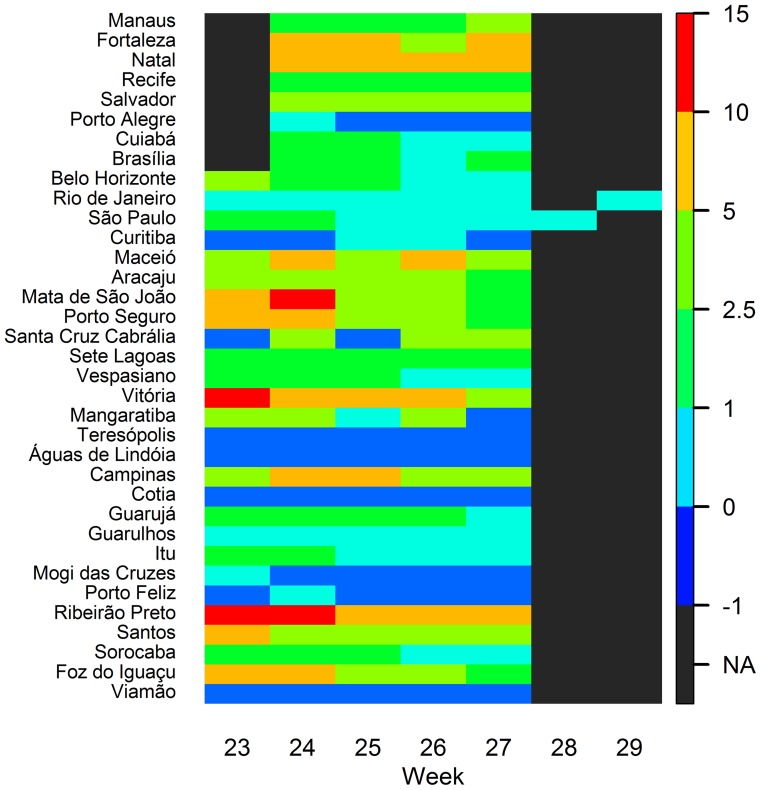
Estimated incidence rates (/100,000) for each city per week of the World Cup based on the 2014 percentile (P2014). Country teams will arrive one week before the games start. During the last two weeks of the tournament, the only games will be the semi-finals in Sao Paulo (week 28) and the finals in Rio de Janeiro (week 29).

Estimated IR's during the World Cup weeks varied over time due to strong seasonality during past dengue epidemics (**Figure 3**). Expected IR's were consistently high (>5/100,000) throughout the World Cup weeks for Natal and Ribeirão Preto. For many other cities, IR's dropped after week 24 or 25 or were consistently low below 5/100,000.

**Figure 4:**
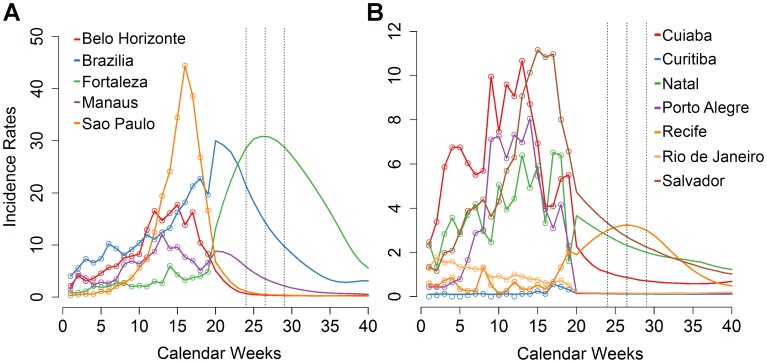
Forecasted weekly incidence rates for 2014 based on an Emperical Bayes model. For each game city, the forecasted IR's are shown separately for cities with (**A**) high, and (**B**) low forecasts. Incidence rates of the first 19 weeks were observed and the remaining weeks are projected forecast values.

The EB models for the 12 game cities resulted in forecasted IR's similar to the PR method except for the cities of Fortaleza and Brasilia (**Table 2**). This difference resulted from the possibility of epidemic time shifting in the EB models that predicted sustained or increasing transmission during the coming weeks in these cities (**Figures 4** **and S3**). Based on EB, the highest IR's during round one were expected in Fortaleza (30.0, 95%CI: 14.4–37.7), followed by Brasilia (18.4, 95%CI: 17.2–19.6). For most cities, forecasted IR's remained similar or dropped slightly during the second round (**Figures 4** **and S3**).

**Table 2:** Average weekly incidence rates during the 2014 World Cup estimated by the Empirical Bayes model.

	Round 1			Round 2		
	Mean	95% CI*		Mean	95%CI*	
Belo Horizonte	0.47	0.10	0.96	0.26	0.10	0.72
Brasilia	18.37	17.18	19.55	11.45	10.33	12.58
Cuiaba	0.85	0.10	2.11	0.60	0.10	1.80
Curitiba	0.11	0.10	0.19	0.11	0.10	0.16
Fortaleza	29.95	14.43	37.65	29.86	25.08	41.77
Manaus	4.79	4.10	5.87	2.64	2.00	3.35
Natal	2.57	1.24	7.02	2.12	0.68	7.58
Porto Alegre	0.12	0.10	0.24	0.12	0.10	0.25
Recife	3.12	1.24	4.92	3.13	1.32	4.74
Rio Janeiro	0.13	0.10	0.27	0.13	0.10	0.25
Salvador	3.06	1.85	6.56	2.42	1.22	5.55
Sao Paulo	0.81	0.47	1.15	0.40	0.10	0.73

*95% credible interval.

### Estimated dengue risk per visiting country

**Figure 5:**
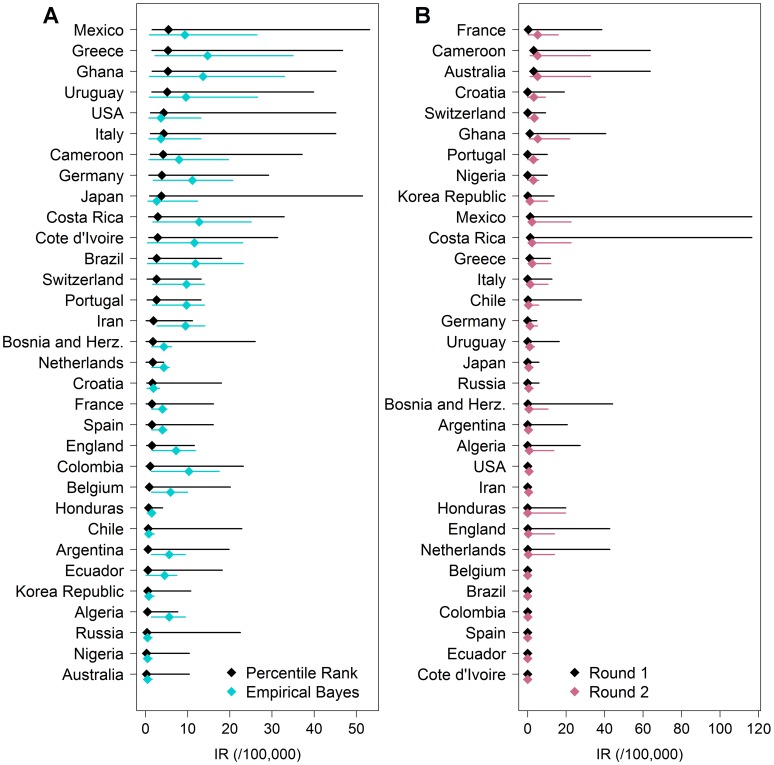
Estimated weekly incidence rates averaged per visiting country. Estimates are shown for (**A**) tourists and (**B**) teams during the World Cup. Estimates for tourists only included round one and were based on the PR (black) and EB (blue) method. Uncertainty around estimated IR's is shown as lines from the 10^th^ percentile to the maximum of the 2001–2013 distribution (PR) and from the 2.5% to 97.5% Bayes credible limits (EB). Estimates for country teams were based on the PR method only.

We computed the weekly IR's expected during round one of the World Cup per country of origin for tourists and teams as the average across their three game cities (**Figure 5A**). The EB method resulted in higher estimates for some countries consistently with the higher risk estimates for Fortaleza and Brasilia. The highest IR's were expected for Mexico at 5.4 cases/100,000/week (PR) and for Cote d'Ivoire at 17.1 cases/100,000/week (EB). We also estimated the IR's for round one and two per country team residing in basecamp cities using the PR method (**Figure 5B**). The highest IR was expected for team France with 11.0 cases/100,000, followed by Cameroon and Australia with 9.1 cases/100,000. Teams with the lowest expected IR's were Colombia, Ecuador, Spain, and Cote d'Ivoire. For all teams, IR's during round two were similar to round one.

### Expected number of dengue cases per country

**Figure 6:**
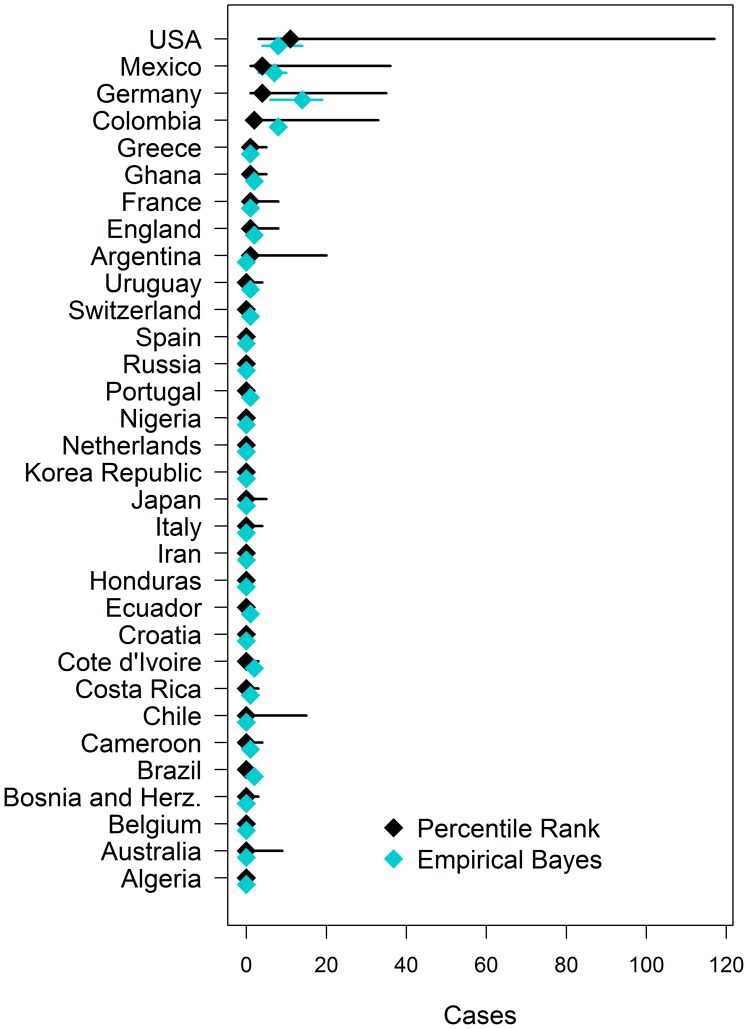
The estimated number of cases among country visitors during round one of the World Cup based on current ticket allocations. Estimates **are shown as diamonds** and uncertainty as lines for the PR (black) and EB (blue) methods. For the PR method, uncertainty limits are the 10^th^ percentile and maximum of the 2001–2013 distribution and for the EB method, these limits are the 95% Bayes credible interval.

Based on current ticket allocations we estimated the expected number of cases among tourists and teams (**Figure 6**). For this, we assumed that all 600,000 tourists will visit games of their respective countries during round one and that they will stay on average for two weeks. The mean number of expected symptomatic dengue cases ranged from 26 to 53 symptomatic cases of dengue on the PR method (P10-Max.: 5–334 cases) and the EB method (95%CI: 30–77) respectively. We expect the most cases among tourists from Germany, the United States, Mexico, and Colombia ranging between 4 and 14 cases for each country depending on the estimation method. We conducted a sensitivity analysis and ranged the number of visitors from 100,000 to 800,000 leading to proportionate numbers of expected dengue cases (**Figure S4**). If 100,000 visitors would attend the World Cup, a total of 4–5 cases would be expected. If the number of visitors would exceed current expectations to 800,000, the number of symptomatic cases would be between 38 and 70 based on the PR and EB method respectively. No dengue cases would be expected among team players due the small number of exposed (23 per team). Even when we increased the team size to 500 (for example including staff, family, and media), the expected number of cases would only be two in the worst case scenario.

## Discussion

We estimated the risk of dengue among tourists and teams during the World Cup games in 2014 using detailed dengue surveillance data from previous years (2001–2013). Except for a few cities (Fortaleza, Belo Horizonte, and Brasilia), the estimated risk for tourists and teams was low. Concern about dengue and other diseases during mass gatherings in general and the Brazil 2014 World Cup in particular have been raised by others [10],[13],[14],[16]–[18]. In the past, outbreaks of predominantly diarrheal and respiratory diseases have been reported during these mass gatherings and the risk of increased DENV transmission during such events may be a real possibility [9],[10],[14]. Recent studies have estimated the risk of dengue in World Cup game cities using varying methodology. One group used spatiotemporal hierarchical Bayesian modeling with climate, demographic, and geographic factors as covariates to predict a high risk of dengue in Recife, Fortaleza, and Natal (>300 cases/100,000/month), a medium risk (100–300) in 4 cities and a low risk (<100) in 5 cities including Brasilia [19]. Our estimates based on DENV transmission in the first 19 weeks of 2014 are different from these and predict a low risk for all cities (<100/100,000/month) except for Fortaleza (120/100,000/month, EB method). This difference is likely due to the low DENV transmission in 2014 compared to previous years. Another group used the force of infection measured from 2010–2013 data to predict that 33 symptomatic cases could be expected (3–59) among 600,000 foreign visitors with the most cases occurring in Rio de Janeiro (11), Fortaleza (10), and Natal (6) [20]. Our estimates are in line with this prediction but we did not estimate as many cases in Rio de Janeiro due to the very low IR expected in this city. The 2014 World Cup in Brazil is one of the first events for which a range of predictions have been made by different groups using varying methodologies. Testing their accuracy after the World Cup will lead to improvements in overall methods and better prediction of disease transmission for future events.

The risk of dengue in World Cup cities was highly dependent on seasonality. The strength of the seasonal pattern was different across cities ranging from elevated transmission year-round in cities such as Natal and Fortaleza to strong epidemic peaks and very low transmission afterwards in cities such as Belo Horizonte, São Paulo, and Rio de Janeiro. Differences in risk estimates between prediction methods were partly due to different assumptions on seasonality. Some methods based predictions for World Cup weeks on only the same weeks in previous years (PR and Massad et. al. [20]) while other methods allowed shifting in epidemic timing and delayed epidemic peaks (EB). The possibility of a delayed peak in the EB models resulted in higher risk estimates for the cities Fortaleza and Brasilia compared to the PR method. In addition to annual seasonality, multiannual variation in dengue epidemics also determined the risk of dengue during the World Cup. Dengue epidemics vary in magnitude from year to year characterized by non-stationary multiannual seasonal patterns that have been described previously [21],[22]. Despite the uncertainty caused by multiannual DENV transmission dynamics, all studies agree that the 2014 epidemic will be smaller compared to previous years due to immunity provided by the large 2013 epidemic and no changes in circulating DENV serotypes [20].

Host immunity is another major determinant of the risk of symptomatic dengue disease. In naïve hosts, DENV infection typically causes mild or no symptoms whereas secondary infection with a heterologous DENV serotype can cause severe disease or death [3]. There will be substantial heterogeneity in the DENV immune status among World Cup visitors. Many tourists will come from neighboring countries with endemic DENV circulation such as Colombia, Costa Rica, Honduras, and Ecuador. Many others will come from countries with no DENV transmission. DENV exposure also varies largely within the Brazil population. In endemic cities such as Recife, about 80% of the population acquired immunity to all DENV serotypes by the age of 20 [23]. Visitors from low transmission areas within or outside Brazil may have been exposed to only one serotype and could be at risk of severe disease during secondary exposure during the games. Many Brazilians however may have no immunity at all. In addition, most visitors will be adults with higher levels of immunity compared to children. Lack of data on previous dengue exposure among World Cup visitors has limited the precision of risk estimates.

We estimated that on average between 26 and 53 symptomatic dengue cases will occur among 600,000 tourists visiting Brazil during the World Cup. This number ranged from a low of 4 to a high of 334 in the worst case scenario. Although slightly different numbers were provided by the PR and EB models, the total number of expected cases remained low. Based on game schedules, the highest number of cases would be expected among tourists from Germany, the US, Mexcio, and Colombia. Data on the exact number of visitors expected are sparse and when we ranged the number of possible visitors from a low of 100,000 to a high of 800,000, we found a total of 4–5 and 38–70 cases respectively. These numbers of symptomatic dengue cases are an underestimate of the number of infections that can also occur asymptomatically. The estimated number of cases highly depended on the duration of stay among tourists. Based on previous studies, we assumed an average duration of stay of two weeks [16]. If tourists would stay longer, the number of cases would increase proportionately. The number of cases also depended on the location of tourists. Because it will remain unknown where each country team will play until completion of round one, we assumed that all tourists would visit during round one. Given that IR's are similar or lower during round two, we don't expect this assumption to affect the accuracy of our estimates. We also assumed that tourists will visit only one game of their country team and will not follow their country to all games due to the extensive travel and cost involved to do this. Data on the numbers of foreign visitors and their travel schedules from previous World Cups would greatly improve these estimates but was unfortunately not available.

The risk posed to local dengue transmission by the influx of susceptible hosts during the World Cup will likely be low. Given the 7 days intrinsic (human) and up to 14 days extrinsic (mosquito) incubation period of the DENV, local transmission must be already ongoing for tourists to be infected during their two week stay. In addition, high levels of population immunity among the local population due to the 2013 outbreak will reduce transmission. If infected tourists would develop symptoms of clinical dengue, this would occur most likely upon return to their home countries. In the absence of a vaccine, the Brazil health authorities will continue vector control and case detection in high-risk areas such as Fortaleza. Tourists can also reduce their risk of dengue by staying in airconditioned accommodation and by applying repellants. The Brazil health authorities should coordinate with World Cup organizers to provide information on local medical care facilities in case tourists do experience symptoms. Physicians in tourist countries of origin should be aware of the possibility of dengue in returning travelers, in particular those from high risk cities. In general however, we do not expect many dengue cases among tourists given the timing of the World Cup and the low transmission rates so far in 2014 compared to previous years. Quantitative risk estimates for disease transmission during mass gatherings by multiple groups using different methodology should be done routinely, leading to increasingly more accurate predictions and better disease preparedness and response.

## Supporting Information

Figure S1 **Percentile of 2014 weekly IR's on distributions of previous years.** For each game city, the percentile of 2014 weeks 1 to 19 on the 2001–2013 distributions for the same weeks is shown (black) with some x-axis random variation to see the individual points. The average percentile weighted by the week number is shown in green (P2014).
(PDF)

Figure S2 **Probability distribution of average log10 incidence rates (/100,000) in game cities of the 2001–2013 period during the weeks of World Cup round one (teams: R1T and tourists: R1G) and round two (R2) of the 2014 Word Cup.** For each period, the 10^th^ percentile (P10), P2014 and maximum are indicated.
(PDF)

Figure S3 **Forecasted log10 incidence rates (/100,000) in game cities per week of 2014 and 95% credible intervals based on an Empirical Bayes model.** Incidence rates for the first 19 weeks were observed values and for the remaining weeks are forecasts. The two rounds of the World Cup are indicated in yellow (round one) and green (round two).
(PDF)

Figure S4 **Estimated number of cases for various numbers of foreign tourists.** The expected number of symptomatic dengue cases was estimated for the number of visitors ranging from 100,000 to 800,000 while keeping the proportion of visitors per country constant. Per country, the number of visitors is shown for varying number of total visitors (in color) using the (**A**) percentile rank method and (**B**) the Empirical Bayes method.
(PDF)

Table S1 Country teams and their game* and basecamp locations.
(PDF)

Table S2 **Number of allocated tickets per country.**
(PDF)

Text S1 **Detailed description of the Empirical Bayes prediction model.**
(PDF)
