# Supplementary material for: Risk of Dengue for Tourists and Teams during the World Cup 2014 in Brazil
Source: PLoS Negl Trop Dis. 2014 Jul 31;8(7):e3063. doi: 10.1371/journal.pntd.0003063 (PMC4120682; doi:10.1371/journal.pntd.0003063)
Supplement: Figure S1 — Percentile of 2014 weekly IR's on distributions of previous years. For each game city, the percentile of 2014 weeks 1 to 19 on the 2001–2013 distributions for the same weeks is shown (black) with some x-axis random variation to see the individual points. The average percentile weighted by the week number is shown in green (P2014). (PDF) [file pntd.0003063.s001.pdf]

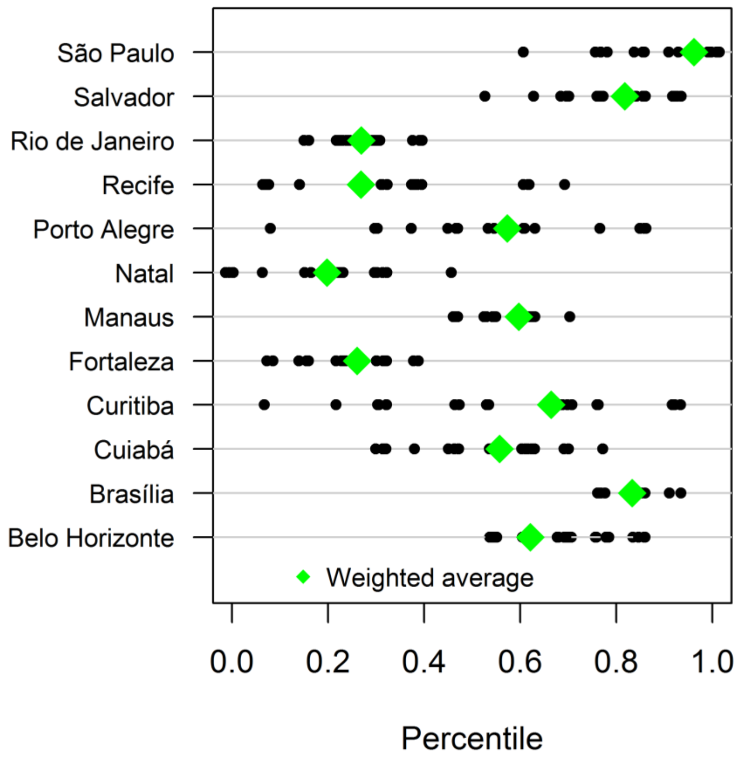

Figure S1, Percentile of 2014 weekly IR's on distributions of previous years. For each game city, the percentile of 2014 weeks 1 to 19 on the 2001-2013 distributions for the same weeks is shown (black) with some x-axis random variation to see the individual points. The average percentile weighted by the week number is shown in green (P2014).
