# Supplementary material for: Risk of Dengue for Tourists and Teams during the World Cup 2014 in Brazil
Source: PLoS Negl Trop Dis. 2014 Jul 31;8(7):e3063. doi: 10.1371/journal.pntd.0003063 (PMC4120682; doi:10.1371/journal.pntd.0003063)
Supplement: Figure S2 — Probability distribution of average log10 incidence rates (/100,000) in game cities of the 2001–2013 period during the weeks of World Cup round one (teams: R1T and tourists: R1G) and round two (R2) of the 2014 Word Cup. For each period, the 10th percentile (P10), P2014 and maximum are indicated. (PDF) [file pntd.0003063.s002.pdf]

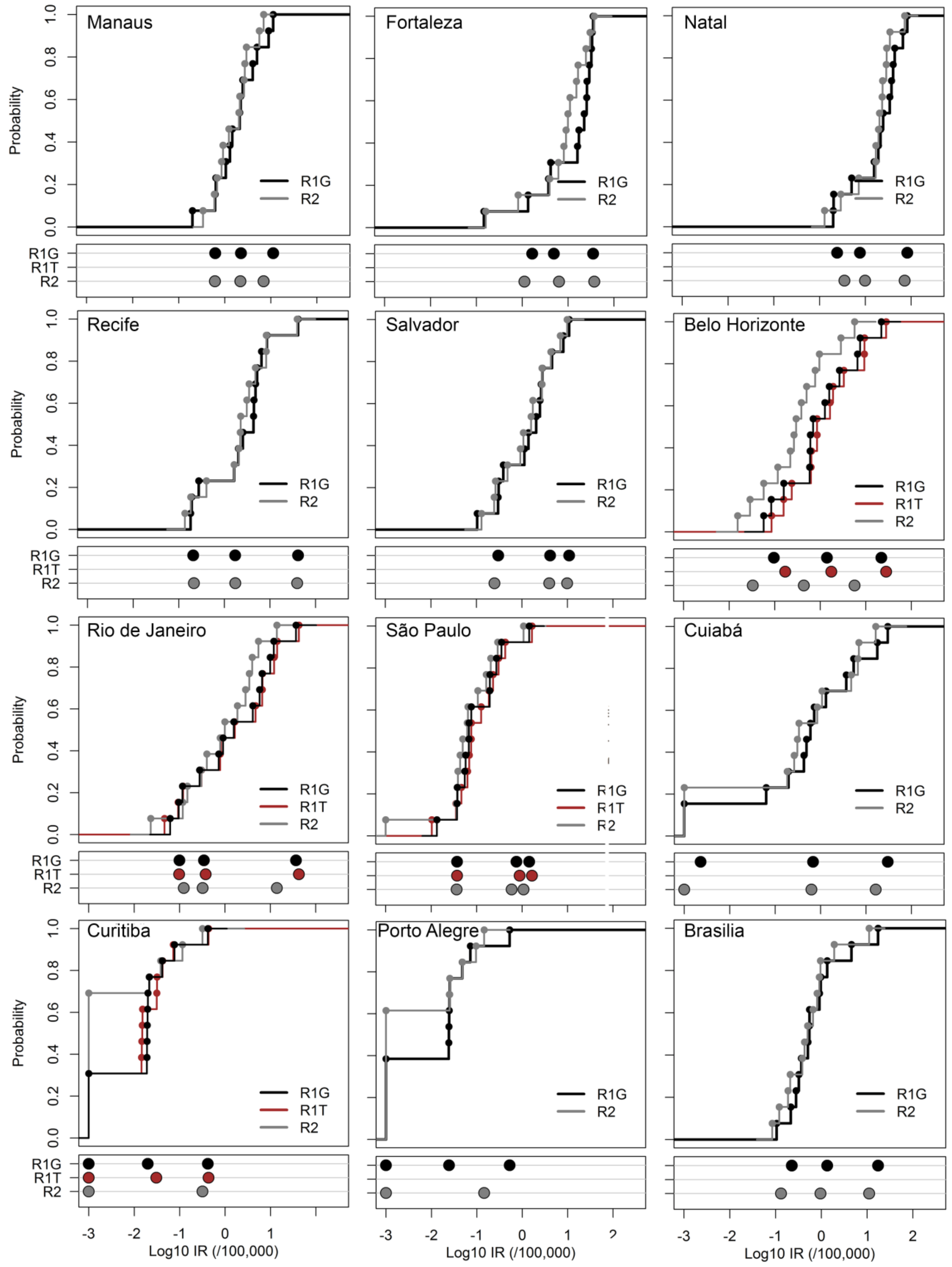

Figure S2. Probability distribution of average log10 incidence rates (/100,000) in game cities of the 2001-2013 period during the weeks of World Cup round one (teams: R1T and tourists: R1G) and round two (R2) of the 2014 Word Cup. For each period, the 10th percentile (P10), P2014 and maximum are indicated.
