# Supplementary material for: Risk of Dengue for Tourists and Teams during the World Cup 2014 in Brazil
Source: PLoS Negl Trop Dis. 2014 Jul 31;8(7):e3063. doi: 10.1371/journal.pntd.0003063 (PMC4120682; doi:10.1371/journal.pntd.0003063)
Supplement: Figure S3 — Forecasted log10 incidence rates (/100,000) in game cities per week of 2014 and 95% credible intervals based on an Empirical Bayes model. Incidence rates for the first 19 weeks were observed values and for the remaining weeks are forecasts. The two rounds of the World Cup are indicated in yellow (round one) and green (round two). (PDF) [file pntd.0003063.s003.pdf]

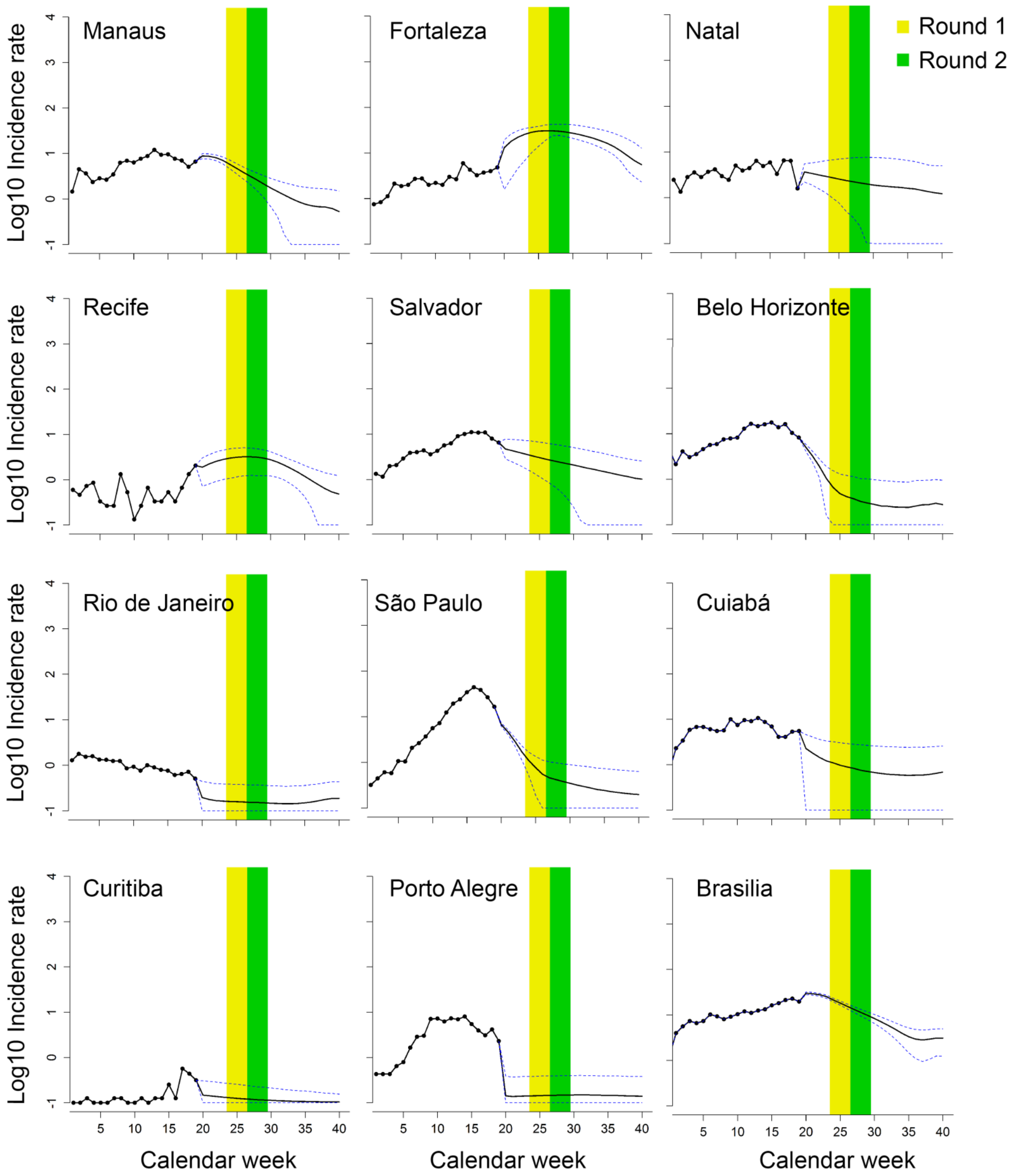

Figure S3. Forecasted log10 incidence rates (/100,000) in game cities per week of 2014 and 95% credible intervals based on an Empirical Bayes model. Incidence rates for the first 19 weeks were observed values and for the remaining weeks are forecasts. The two rounds of the World Cup are indicated in yellow (round one) and green (round two).
