# Supplementary material for: Risk of Dengue for Tourists and Teams during the World Cup 2014 in Brazil
Source: PLoS Negl Trop Dis. 2014 Jul 31;8(7):e3063. doi: 10.1371/journal.pntd.0003063 (PMC4120682; doi:10.1371/journal.pntd.0003063)
Supplement: Figure S4 — Estimated number of cases for various numbers of foreign tourists. The expected number of symptomatic dengue cases was estimated for the number of visitors ranging from 100,000 to 800,000 while keeping the proportion of visitors per country constant. Per country, the number of visitors is shown for varying number of total visitors (in color) using the (A) percentile rank method and (B) the Empirical Bayes method. (PDF) [file pntd.0003063.s004.pdf]

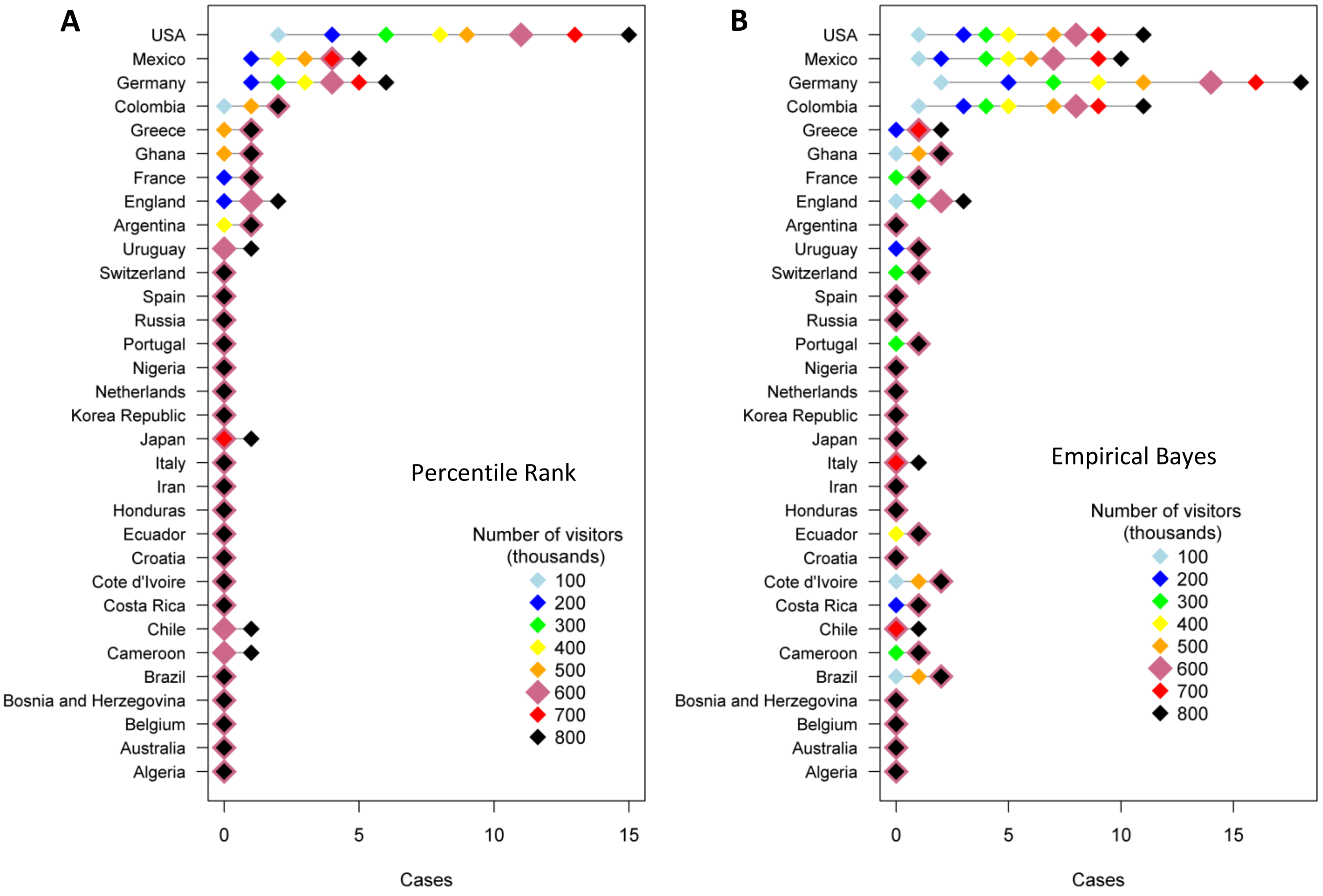

Figure S4. Estimated number of cases for various numbers of foreign tourists. The expected number of symptomatic dengue cases was estimated for the number of visitors ranging from 100,000 to 800,000 while keeping the proportion of visitors per country constant. Per country, the number of visitors is shown for varying number of total visitors (in color) using the (**A**) percentile rank method and (**B**) the Empirical Bayes method.
