# Supplementary material for: Risk of Dengue for Tourists and Teams during the World Cup 2014 in Brazil
Source: PLoS Negl Trop Dis. 2014 Jul 31;8(7):e3063. doi: 10.1371/journal.pntd.0003063 (PMC4120682; doi:10.1371/journal.pntd.0003063)
Supplement: Table S1 — Country teams and their game* and basecamp locations. (PDF) [file pntd.0003063.s005.pdf]

Table S1, Country teams and their game* and basecamp locations

**Country team**	**Game 1**	**Game 2**	**Game 3**	**Basecamp**
Algeria	Curitiba	Belo Horizonte	Porto Alegre	Sorocaba
Argentina	Belo Horizonte	Porto Alegre	Rio de Janeiro	Vespasiano
Australia	Curitiba	Cuiabá	Porto Alegre	Vitória
Belgium	Rio de Janeiro	Belo Horizonte	São Paulo	Mogi das Cruzes
Bosnia and Herzegovina	Salvador	Cuiabá	Rio de Janeiro	Guarujá
Cameroon	Manaus	Natal	Brasília	Vitória
Chile	Rio de Janeiro	Cuiabá	São Paulo	Belo Horizonte
Colombia	Cuiabá	Brasília	Belo Horizonte	Cotia
Costa Rica	Fortaleza	Belo Horizonte	Recife	Santos
Cote d'Ivoire	Recife	Fortaleza	Brasília	Águas de Lindóia
Croatia	São Paulo	Manaus	Recife	Mata de São João
Ecuador	Curitiba	Brasília	Rio de Janeiro	Viamão
England	São Paulo	Manaus	Belo Horizonte	Rio de Janeiro
France	Rio de Janeiro	Salvador	Porto Alegre	Ribeirão Preto
Germany	Recife	Fortaleza	Salvador	Santa Cruz Cabrália
Ghana	Fortaleza	Natal	Brasília	Maceió
Greece	Natal	Fortaleza	Belo Horizonte	Aracaju
Honduras	Porto Alegre	Curitiba	Manaus	Porto Feliz
Iran	Belo Horizonte	Curitiba	Salvador	Guarulhos
Italy	Recife	Natal	Manaus	Mangaratiba
Japan	Cuiabá	Natal	Recife	Itu
Korea Republic	São Paulo	Cuiabá	Porto Alegre	Foz do Iguaçu
Mexico	Natal	Fortaleza	Recife	Santos
Netherlands	Porto Alegre	Salvador	São Paulo	Rio de Janeiro
Nigeria	Porto Alegre	Cuiabá	Curitiba	Campinas
Portugal	Manaus	Brasília	Salvador	Campinas
Russia	Cuiabá	Rio de Janeiro	Curitiba	Itu
Spain	Salvador	Rio de Janeiro	Curitiba	Curitiba
Switzerland	Manaus	Brasília	Salvador	Porto Seguro
Uruguay	São Paulo	Natal	Fortaleza	Sete Lagoas
USA	Recife	Manaus	Natal	São Paulo

* Game locations are only known for round one (qualifying round).
