# Supplementary material for: Risk of Dengue for Tourists and Teams during the World Cup 2014 in Brazil
Source: PLoS Negl Trop Dis. 2014 Jul 31;8(7):e3063. doi: 10.1371/journal.pntd.0003063 (PMC4120682; doi:10.1371/journal.pntd.0003063)
Supplement: Table S2 — Number of allocated tickets per country. (PDF) [file pntd.0003063.s006.pdf]

Table S2, Number of allocated tickets per country [1]

Country	Tickets
USA	125,465
Colombia	60,231
Germany	55,666
Argentina	53,809
England	51,222
Australia	40,446
France	34,971
Chile	32,189
Mexico	30,238
Total	484,237

References

1. International Federation of Association Football (2014) 2.3 million tickets for the 2014 FIFA World Cup already allocated to fans. 21 Febr. Available: http://www.fifa.com/worldcup/news/y=2014/m=2/news=million-tickets-for-the-2014-fifa-world-cuptm-already-allocated-fans-2281407.html. Accessed 3 April 2014.
