# Supplementary material for: Risk of Dengue for Tourists and Teams during the World Cup 2014 in Brazil
Source: PLoS Negl Trop Dis. 2014 Jul 31;8(7):e3063. doi: 10.1371/journal.pntd.0003063 (PMC4120682; doi:10.1371/journal.pntd.0003063)
Supplement: Text S1 — Detailed description of the Empirical Bayes prediction model. (PDF) [file pntd.0003063.s007.pdf]

Supplemental Text 1

For the Empirical Bayes approach, we used the following statistical model of the time series of confirmed dengue cases for a particular municipality over the past twelve observed seasons:

$$Y^s = f^s + \epsilon^s, \quad \epsilon^s \sim \mathcal{N}(0, \sigma_s^2 I) \quad f^s \sim F$$
$$s = 1, 2, \ldots, 12$$
$$Y^s, f^s, \epsilon^s \in \mathbb{R}^{52}$$

Y^s: observed epidemic trajectory for season s
f^s: true underlying epidemic curve for season s (Denote i'th entry of f^s as $f^s(i)$)
ϵ^s: vector of noise (measurement errors) for season s
σ_s^2: variance of the noise ϵ^s

Our choice of the prior distribution of curves F is indexed by a triplet (f, μ, δ) where f represents a particular season's underlying curve, μ represents the scaling of its attained values, and δ represents the shifting of the location of the curve f. We assumed that f followed a discrete uniform distribution over f_0^s, Unif$\{f_0^s, s = 1, \ldots, N\}$, where f_0^s were historical trajectories Y^s from season s that was smoothed by quadratic trend filtering. We assumed that μ and δ follows a uniform distribution within a fixed range, $U(\mu_m, \mu_M)$ for μ and $U(\delta_m, \delta_M)$ for δ. These distributions characterize transformations of past season's curves. The effect of these transformations is to shift and scale f to have a peak height and peak location within a reasonable range of itself, with $\mu_m = -2, \mu_M = 2$ for a time shift (shift in index) and $\delta_m = .75, \delta_M = 1.25$ for a vertical scale. A variation of this model would be to transform the drawn curves to have peak heights and locations corresponding to a randomly drawn value within the historical range. We found this however to be an unreasonable assumption for an epidemic pattern with vastly different scales across seasons.

Described generatively: we randomly drew $\hat{f}_0$ from $\{f_0^s, \ s = 1, 2, \ldots, 12\}$, μ from $U(\mu_m, \mu_M)$, and δ from $U(\delta_m, \delta_M)$ to form a sample curve from F, $g_0 \in \mathbb{R}^{52}$, whose j'th entry is defined as $g_0(j) = \delta \hat{f}_0(j + \text{argmax}_i \hat{f}_0 - \mu)$. Because of the analytical difficulty of drawing from the posterior distribution directly, we used a modified (for computational efficiency reasons) importance sampling in which we drew sample curves from a discretized version of the prior distribution and assigned likelihood-based importance weights to each sample. These weights are proportional to the likelihood of a curve drawn from the prior, given the observed data in the current (2014) season. The statistical model used in the likelihood calculation for the current season trajectory that we used in the importance sampling weight calculation is identical with that of past seasons:

$$Y^{13} = f^{13} + \epsilon^{13}, \epsilon \sim \mathcal{N}(0, \sigma_{13}^2 I)$$
$$Y^{13}, f^{13}, \epsilon^{13} \in \mathbb{R}^{52}$$

where for σ_{13}^2 we assumed the same observed noise level of season s that the curve f^{13} originated from. Since we only observed 2014 (season 13) IR's up to the week 19, we only used information from these weeks as data: $Y^{13}(i) = f^{13}(i) + \epsilon^{13}(i), \ i = 1, \ldots, 19$. Since we treated each time point as independent, the likelihood was calculated as a product of 19 univariate Gaussian densities.

The goal was to make inference about the noise-injected trajectory Y^{13} of the current season, or functions of it, $g(Y^{13})$. In this paper, we forecasted the entire upcoming trajectory of this season (weeks 20-52) and some specific forecasting targets, which are functions of this trajectory: the forecasted IR at each week of the World Cup period $g(Y^{13}) = Y^{13}(i), i = 24, 25, \cdots, 29$ and the average IR for round 1 and 2 of the World Cup period, $g(Y^{13}) = \frac{1}{3}\sum_{i \in \{24,25,26\}} Y^{13}(i)$ and $g(Y^{13}) = \frac{1}{3}\sum_{i \in \{27,28,29\}} Y^{13}(i)$. We obtained these forecasting targets by applying the above functions $g(\cdot)$ to the posterior sample curves obtained by importance sampling, thus obtaining a posterior sample

of the forecasting targets. From this we produced point estimates of the forecasting targets, and uncertainty measures such as Bayesian posterior intervals for forecasting targets and posterior bands for the upcoming season's IR trajectory.
